# Higher frequency but random distribution of *EGFR* mutation subtypes in familial lung cancer patients

**DOI:** 10.18632/oncotarget.10715

**Published:** 2016-07-19

**Authors:** Kuo-Hsuan Hsu, Jeng-Sen Tseng, Chih-Liang Wang, Tsung-Ying Yang, Chien-Hua Tseng, Hsuan-Yu Chen, Kun-Chieh Chen, Chi-Ren Tsai, Cheng-Ta Yang, Sung-Liang Yu, Kang-Yi Su, Chong-Jen Yu, Chao-Chi Ho, Te-Chun Hsia, Ming-Fang Wu, Kuo-Liang Chiu, Chien-Ming Liu, Pan-Chyr Yang, Jeremy J.W. Chen, Gee-Chen Chang

**Affiliations:** ^1^ Institute of Biomedical Sciences, National Chung Hsing University, Taichung, Taiwan; ^2^ Division of Critical Care and Respiratory Therapy, Department of Internal Medicine, Taichung Veterans General Hospital, Taichung, Taiwan; ^3^ Division of Chest Medicine, Department of Internal Medicine, Taichung Veterans General Hospital, Taichung, Taiwan; ^4^ Faculty of Medicine, School of Medicine, National Yang-Ming University, Taipei, Taiwan; ^5^ Department of Thoracic Medicine, Chang Gung Memorial Hospital, Taoyuan, Taiwan; ^6^ College of Medicine, Chang Gung University, Taoyuan, Taiwan; ^7^ Institute of Epidemiology and Preventive Medicine, National Taiwan University, Taipei, Taiwan; ^8^ Department of Critical Care Medicine, Taichung Veterans General Hospital, Taichung, Taiwan; ^9^ Institute of Statistical Science, Academia Sinica, Taipei, Taiwan; ^10^ College of Medicine, National Taiwan University, Taipei, Taiwan; ^11^ College of Life Science, National Taiwan University, Taipei, Taiwan; ^12^ Department of Pediatrics, Taichung Veterans General Hospital, Taichung, Taiwan; ^13^ Institute of Molecular Biology, National Chung Hsing University, Taichung, Taiwan; ^14^ Department of Respiratory Therapy, College of Medicine, Chang Gung University, Taoyuan, Taiwan; ^15^ Department of Clinical Laboratory Sciences and Medical Biotechnology, College of Medicine, National Taiwan University, Taipei, Taiwan; ^16^ Department of Laboratory Medicine, National Taiwan University Hospital, Taipei, Taiwan; ^17^ Center of Genomic Medicine, National Taiwan University, Taipei, Taiwan; ^18^ Department of Pathology and Graduate Institute of Pathology, College of Medicine, National Taiwan University, Taipei, Taiwan; ^19^ Center for Optoelectronic Biomedicine, College of Medicine, National Taiwan University, Taipei, Taiwan; ^20^ Division of Pulmonary Medicine, Department of Internal Medicine, National Taiwan University Hospital and National Taiwan University College of Medicine, Taipei, Taiwan; ^21^ Department of Internal Medicine, China Medical University Hospital, Taichung, Taiwan; ^22^ Department of Respiratory Therapy, China Medical University, Taichung, Taiwan; ^23^ Divisions of Medical Oncology and Pulmonary Medicine, Department of Internal Medicine, Chung Shan Medical University Hospital, Taichung, Taiwan; ^24^ School of Medicine, Chung Shan Medical University, Taichung, Taiwan; ^25^ Division of Chest Medicine, Department of Internal Medicine, Taichung Tzu-Chi Hospital, Taichung, Taiwan; ^26^ School of Post-Baccalaureate Chinese Medicine, Tzu Chi University, Hualien, Taiwan

**Keywords:** familial lung cancer, epidermal growth factor receptor (EGFR), non-small cell lung cancer, YAP1

## Abstract

Despite the advancement of *epidermal growth factor receptor* (*EGFR*) inhibitors in lung cancer therapy, it remains unclear whether *EGFR* mutation status in familial lung cancers is different from that of sporadic cases. In this multicenter retrospective study, we compared both the *EGFR* mutation frequency and patterns between familial and sporadic cases. The results explored that family history of lung cancer is an independent predictor for higher *EGFR* mutation rate in 1713 lung adenocarcinoma patients (Odd ratio 1.68, 95% CI 1.06–2.67, *P* = 0.028). However, the distribution of *EGFR* mutation subtypes was similar to that of sporadic cases. Part of our study involved 40 lung cancer families with at least 2 tumor tissues available within each single family (*n* = 88) and there was no familial aggregation pattern in *EGFR* mutation subtypes. There were two families harboring the *YAP1* R331W germline risk allele and *EGFR* mutation statuses among *YAP1* family members also varied. These phenomena may hint at the direction of future research into lung carcinogenesis and *EGFR* mutagenesis.

## INTRODUCTION

Lung cancer is the leading cause of cancer-related death worldwide [1]. Although smoking is a well-known risk factor for lung cancer, about 25% of lung cancer cases in the world occur in never smokers, which are more common in East Asians [2, 3]. In Taiwan, more than 50% of all lung cancer patients are never smokers [4]. This phenomenon has been investigated in a number of studies, which have explored lung cancer risk factors other than smoking, including the role of genetic variations and heredity.

Many studies have proved family history of lung cancer as a risk factor in this disease and demonstrated that familial aggregation of lung cancer does exist after adjusting for smoking behaviors and types of family relatedness [5]. In a pooled analysis from the International Lung Cancer Consortium (ILCCO), individuals with family history of lung cancer in a first-degree relative had a 1.5-fold increased risk of lung cancer after adjusting for other known risk factors, and the association was highest among Asians [6]. Therefore, further research is needed to determine the role of genetic susceptibility in the tumorigenesis of lung cancer.

*Epidermal growth factor receptor (EGFR)* mutation is the most common genetic alteration in East Asians with lung cancer [7, 8]. EGFR-tyrosine kinase inhibitors (TKIs) have emerged as an effective therapy among patients with advanced stage *EGFR*-mutant non-small cell lung cancer (NSCLC), as they result in more favorable outcomes as well as a better quality of life [9–12]. In a study by Gaughan et al. non-smoker NSCLC patients who harbored *EGFR* mutations were shown to have a significantly higher rate of family history of lung cancer as compared with *ALK*- and *KRAS*-mutated cohorts, which suggests that *EGFR* mutations might involve in the heredity of lung cancer [13].

Despite the advancement of *EGFR* inhibitors in lung cancer therapy, it remains unclear whether *EGFR* mutation status in familial lung cancers is different from that of sporadic cases. Herein, we evaluated both the *EGFR* mutation frequency and spectrum in familial lung cancer patients.

## RESULTS

### Participant selection

**Figure 1:**
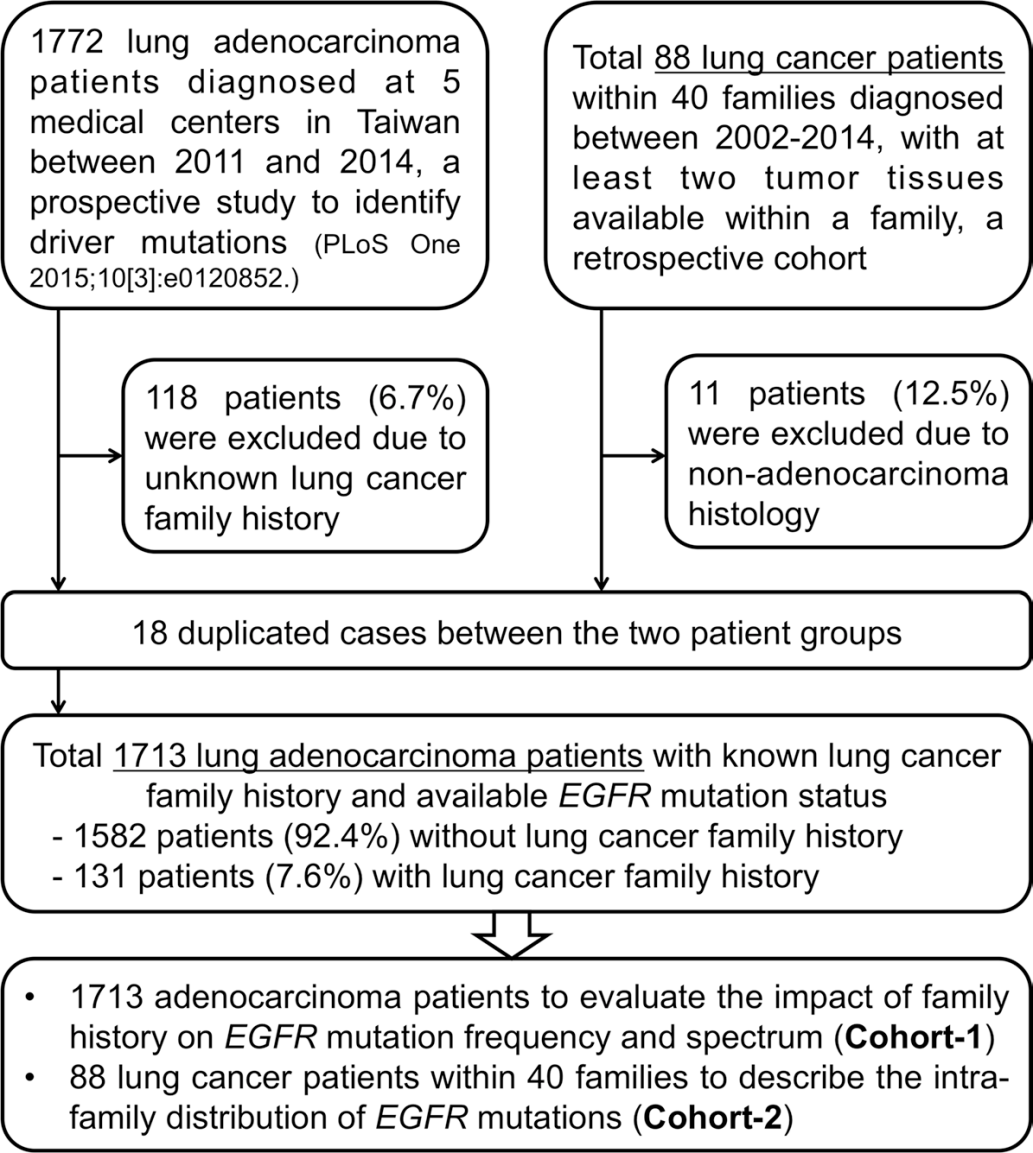
Study participant selection flowchart

The participant selection flowchart is disclosed in Figure 1. With regards to the *EGFR* mutation frequency and spectrum, we only evaluated patients with lung adenocarcinoma to eliminate the confounding effect of different histology. Among the multicenter prospective cohort (*n* = 1772), 118 patients (6.7%) were excluded due to uncertain lung cancer family history. Among the retrospective familial lung cancer cohort (*n* = 88), 11 patients (12.5%) were excluded while analyzing the overall *EGFR* mutation frequency and spectrum due to non-adenocarcinoma histology. There were 18 duplicated cases between the two groups; hence, a total of 1713 lung adenocarcinoma patients were indicated as “Cohort-1” to evaluate the role of lung cancer family history on *EGFR* mutations. Of Cohort-1, 131 patients (7.6%) had lung cancer family history and 1582 patients (92.4%) were sporadic cases.

The retrospective familial lung cancer cohort, including lung cancer patients with at least 2 tumor tissues available within a family, were indicated as “Cohort-2” to evaluate the intra-family distribution of *EGFR* mutations. Herein, we reserved patients with non-adenocarcinoma histology in order to demonstrate the detail characteristics of lung cancer families. Cohort-2 included 40 lung cancer families and a total of 88 patients. Each family in this cohort contained 2 to 5 members with lung cancer and the family relatedness included both parents-offsprings and siblings.

### Characteristics of patients with lung cancer family history

A comparison of the characteristics between lung adenocarcinoma patients with and without lung cancer family history is shown in Table 1. In terms of histology, a patient with atypical adenomatous hyperplasia (AAH) was enrolled because it is thought to be a premalignant lesion of lung adenocarcinoma [14], and the stage of this patient was not applicable. Our results suggested that patients with lung cancer family history were younger (58.7 vs. 63.8 years, *P* < 0.001) and had earlier tumor stage (stage I–IIIA 40.8% vs. 29.7%, *P* = 0.008). There were no statistical significance for gender and smoking status between each group.

**Table 1:** Characteristics between lung adenocarcinoma patients with and without family history of lung cancer (Cohort-1; *n* = 1713)

Characteristics	Total (*n* = 1713)	Without family history (*n* = 1582)	With family history (*n* = 131)	*P* value^a^
**Age, years, mean (SD)**	63.4 (12.7)	63.8 (12.7)	58.7 (12.6)	< 0.001
**Gender, n (%)**				
Male	784 (45.8)	735 (46.5)	49 (37.4)	0.055
Female	929 (54.2)	847 (53.5)	82 (62.6)	
**Smoking, n (%)**				
Non-smokers	1398 (81.6)	1292 (81.7)	106 (80.9)	0.815
Current/former smokers	315 (18.4)	290 (18.3)	25 (19.1)	
**Stage, n (%)^b^**				
I–IIIA	523 (30.5)	470 (29.7)	53 (40.8)	0.008
IIIB–IV	1189 (69.5)	1112 (70.3)	77 (59.2)	
***EGFR* status, n (%)**				
Wild type	757 (44.2)	711 (44.9)	46 (35.1)	0.045
Mutant^c^	956 (55.8)	871 (55.1)	85 (64.9)	
– Exon 21 L858R	– 473 (45.9)	– 435 (46.3)	– 38 (41.3)	0.560
– Exon 19 deletions	– 457 (44.3)	– 415 (44.2)	– 42 (45.7)	
– Others	– 101 (9.8)	– 89 (9.5)	– 12 (13.0)	

SD, standard deviation; EGFR, epidermal growth factor receptor.

a Age by Student's *t* test and the others by Fisher's exact test.

b One patient of the “with family history” group with atypical adenomatous hyperplasia (AAH) was not applicable.

c A total of 1031 mutations were identified among 956 EGFR-mutant patients.

The *EGFR* mutation rate of Cohort-I was 55.8%, which was comparable with that of Asian patients with lung adenocarcinoma [8]. Of the 956 *EGFR*-mutant patients, 55 patients harbored complex mutations; hence, a total of 1031 mutations were identified. Similarly, exon 19 deletions (44.3%) and exon 21 L858R (45.9%) accounted for the major mutation types. Of note, patients with lung cancer family history had a significantly higher *EGFR* mutation rate (64.9% vs. 55.1%, *P* = 0.045) but the *EGFR* mutation spectrum was similar with that of the sporadic cases (*P* = 0.560).

### Lung cancer family history independently predicts a higher EGFR mutation rate

Although univariate analysis suggested a higher *EGFR* mutation rate among patients with lung cancer family history, the demographics between each group were not even. We conducted multivariate logistic regression analysis and propensity score model to estimate the risk of *EGFR* mutation.

The relationship between lung cancer family history and *EGFR* mutation rate is shown in Table 2. In the unadjusted model of unconditional logistic regression analysis (*n* = 1713), there was a significant association between family history of lung cancer and *EGFR* mutation rate (Odds ratio 1.47, 95% CI 1.01–2.14, *P* = 0.042). After adjusting for all variables, lung cancer family history remained a significant predictor of *EGFR* mutations (Odds ratio 1.53, 95% CI 1.02–2.27, *P* = 0.035). In the case of family relatedness, siblings predicted a similar risk of *EGFR* mutations as compared with parents-offsprings (Odds ratio 1.32, 95% CI 0.63–2.75, *P* = 0.466).

**Table 2:** Unconditional and conditional logistic regression analysis of family lung cancer history and *EGFR* mutation frequency among lung adenocarcinoma patients (Cohort-1)

Analysis	Odds ratio	95% CI	*P* value
With vs. Without family history			
**Unconditional (*n* = 1713)**			
Unadjusted	1.47	1.01–2.14	0.042
Adjusted for all variables^a^	1.53	1.03–2.27	0.035
**Conditional (1:2 matching, *n* = 387)**			
Unadjusted	1.86	1.20–2.87	0.005
Adjusted for propensity	1.83	1.19–2.84	0.006
Adjusted for propensity and all variables^a^	1.68	1.06–2.67	0.028

EGFR, epidermal growth factor receptor; CI, confidence interval.

a Include age, gender, smoking status, and stage.

A full non-parsimonious logistic regression model was fit to calculate the propensity score. Kernel density plots of propensity score before and after matching for familial lung adenocarcinoma patients are shown in Supplementary Figure S1A and S1B. In the unadjusted model (1:2 matching, *n* = 387), there was a significant relationship between lung cancer family history and *EGFR* mutation rate (Odds ratio 1.86, 95% CU 1.20–2.87,*P* = 0.005). After adjusting for propensity and all variables, lung cancer family history remained a significant predictor of *EGFR* mutations (Odd ratio 1.83, 95% CI 1.19–2.84, *P* = 0.006 and 1.68, 95% CI 1.06–2.67, *P* = 0.028, respectively). All these results suggested that family history of lung cancer is independently associated with a higher *EGFR* mutation rate.

### Random distribution of EGFR mutations among lung cancer families

As family history of lung cancer was associated with a significantly higher *EGFR* mutation rate, we further evaluated the distribution of mutation subtypes (Table 3). A propensity score with 1:2 matching (*n* = 248) was conducted to estimate the chance of specific *EGFR* mutation types among *EGFR*-mutant lung adenocarcinoma patients with family history of lung cancer. Kernel density plots of propensity score before and after matching for *EGFR*-mutant familial lung adenocarcinoma patients are shown in Supplementary Figure S1C and S1D. The Odds ratios for the chance of G719X, exon 19 deletions, L858R, and other uncommon mutations were 3.33 (95% CI 0.80–13.95), 1.05 (95% CI 0.62–1.80), 0.89 (95% CI 0.52–1.49), and 0.83 (95% CU 0.29–2.37), respectively. All of the *P value*s were not statistically significant. These results suggested that the *EGFR* mutation subtype spectrum of patients with and without lung cancer family history was similar in distribution.

**Table 3:** Conditional logistic regression analysis for mutation subtypes among *EGFR*-mutant lung adenocarcinoma patients with or without family lung cancer history (1:2 matching, *n* = 248)

*EGFR* mutation types	Odds ratio	95% CI	*P* value
**With vs. Without family history**			
Exon 18 G719X	3.33	0.80–13.95	0.100
Exon 19 deletions	1.05	0.62–1.80	0.855
Exon 21 L858R	0.89	0.53–1.49	0.659
Other uncommon mutations	0.83	0.29~2.37	0.732

EGFR, epidermal growth factor receptor; CI, confidence interval.

**Figure 2:**
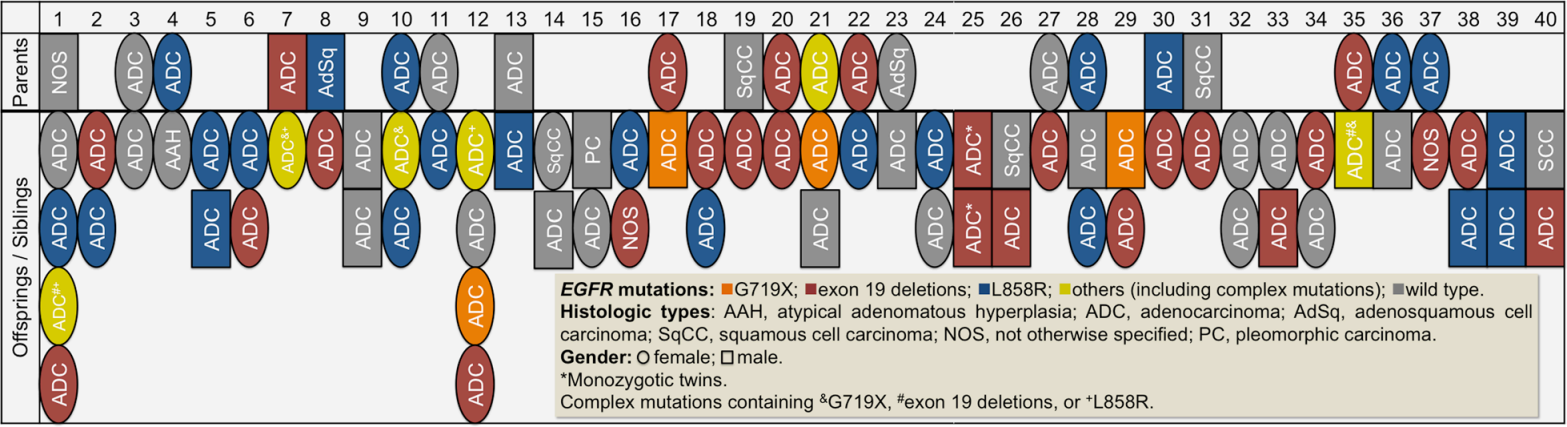
Characteristics and *Epidermal growth factor receptor* (*EGFR*) mutation status of 40 families with at least 2 first relatives with lung cancer in a family (Cohort-2; *n* = 88)

Beyond the *EGFR* mutation status of overall population, we further described the intra-family distribution of *EGFR* mutations. Figure 2 shows detailed information regarding gender, histological types, and the *EGFR* mutation spectrum of the 40 lung cancer families (Cohort-2). Overall, only 10 families (25.0%) had identical *EGFR* mutation status among their family members, including 2 families with exon 19 deletions, 2 families with L858R and 6 families with wild type. In 23 families with pure sibling relatedness, only 7 families (30.4%) had identical *EGFR* mutation status and 4 of them were wild type. Even in 29 families with pure adenocarcinoma histology, only 7 families (24.1%) had identical *EGFR* mutation status. Figure 2 indicates that *EGFR* mutation subtypes within most of the lung cancer families varied and there was no familial aggregation pattern.

Members of Family-25 were monozygotic twins and both of them were diagnosed with lung adenocarcinoma at the same age (58 years), one with RUL lung tumor, stage IV and the other with LLL lung tumor, stage IIIB. Interestingly, both of them harbored exon 19 deletions (1 with Del E746_A750 and 1 with Del E746_S752 ins V).

### Germline YAP1 R331W missense mutation and EGFR mutations

**Figure 3:**
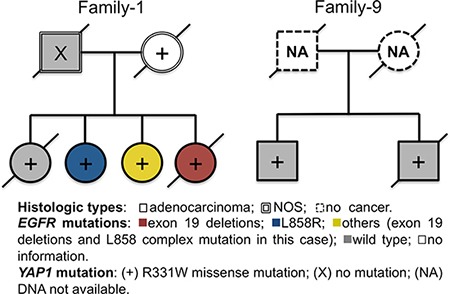
Pedigrees and *Epidermal growth factor receptor* (*EGFR*) mutation status of two families harboring germline *YAP1* R331W missense mutation

Recently, we identified the *YAP1* R331W missense mutation as an allele that predisposes to lung adenocarcinoma in Taiwan with high familial penetrance [15]. In the present study, we also evaluated the association between *YAP1* germline mutation and *EGFR* mutation status among lung cancer families (Cohort-2). There were two families that harbored this germline risk allele (Family-1 and Family-9). The pedigrees and *EGFR* mutation status of the two families are shown in Figure 3. *EGFR* mutation status among these *YAP1*-mutant family members also varied.

## DISCUSSION

In East Asia, there are several unique characteristics of lung cancer, including the predominance of adenocarcinoma histology and a large proportion of never smokers and females [2, 16, 17]. Similarly, these characteristics are clinical factors associated with *EGFR* mutations and better response to EGFR-TKIs therapy [8, 18]. These phenomena suggest directions for further research into the tumorigenesis and pathophysiology of lung cancer. Much remains unknown about the carcinogenetic pathway of *EGFR* mutations despite its identification as a predictive marker for EGFR-TKIs efficacy. As the genetic similarity is supposed to be much higher among first-degree relatives, the major objective of this study was to evaluate the possible link between the presentation of *EGFR* mutations and a family history of lung cancer.

Cancer development is a complex process that involves both genetic and environmental factors [19], which are known to interact. Lung cancer families in our study consisted of both parents-offsprings and siblings. As compared with the general population [7, 8], the diagnostic age of familial lung cancers was younger and the tumor stage was earlier. This might be due to the heightened observation of physicians owing to the familial risk. Indeed, besides the environmental factors, including smoking, many studies have identified family history as a risk factor of lung cancer development [20–22]. In a population-based study using the Swedish Family-Cancer Database, Hemminki et al. showed that 1.7% of lung cancer patients up to age 68 years may be attributable to heredity. The involvement of a high-penetrant recessive gene was suggested in this study because siblings with parents without lung cancer carry a larger familial risk than offsprings of lung cancer parents [21]. Gaughan et al. further showed that the percentage of lung cancer cases with family history of lung cancer was higher in the *EGFR*-mutated versus *EGFR*-wild type NSCLC [13]. These results suggest the role of genetic susceptibility in familial lung cancers.

In the present study, the *EGFR* mutation rate of overall familial lung cancer patients was 64.9%, which was higher than that found in the Asian PIONEER study (51.4%) and our sporadic adenocarcinoma cohort [7, 8]. In a study by Shigematsu et al. lung cancer patients with East Asian ethnicity, including those from the United States and Australia, still had a higher *EGFR* mutation frequency than Caucasian populations [23]. He et al. further showed that NSCLC patients with a family history of cancer, especially lung cancer, had a higher frequency of *EGFR* mutations [24]. These observations suggest that not only the lung carcinogenesis but also the *EGFR* mutagenesis might involve a potential inherited predisposition.

Although previous studies have shown a positive association between family history of lung cancer and *EGFR* mutation prevalence [13, 24], the *EGFR* mutation subtype spectrum among lung cancer families has not been investigated yet. In the present study, each lung cancer family in Cohort-2 consisted of at least two members with this disease and all of them had available tissue for *EGFR* mutation analysis. These data made it possible to explore the intra-family distribution of *EGFR* mutations. Our results showed that only a few families had identical *EGFR* mutation subtypes between family members. Our analysis of lung cancer patients within the same generation, the siblings, also revealed no trend of higher similarity in the spectrum of *EGFR* mutations. This finding indicated that after adjusting for environmental effects, there could be a genetic predisposition and individual susceptibility for different *EGFR* mutation subtypes. In fact, the overall mutation spectrum among familial lung cancer patients, especially the common mutations, was similar to that of sporadic cases. In a case report by Kurahara et al. a father and son, both non-smokers, developed NSCLC and lung adenocarcinoma [25]. However, they harbored different *EGFR* mutation patterns, one with L858R and one with exon 19 deletions. This phenomenon was similar to our observations in most of our lung cancer families. Therefore, we suggest that the distribution of *EGFR* mutation subtypes among lung cancer families was random, rather than showing a pattern of familial aggregation.

In our study, we had a family with monozygotic twin brothers. Although earlier studies did not suggest a significant effect of inherited predisposition on development of lung cancer in twins [26, 27], they remain important candidates in research into the genetic familial effects on *EGFR* mutagenesis because of their genetic similarity. The twin brothers in our study were diagnosed with advanced stage lung cancer at the same age and they shared the same histology and *EGFR* mutation subtype. This observation might not just be coincidental. Further studies involving twins with lung cancer would be useful to evaluate the effects of genetic predisposition on *EGFR* mutations.

Germline mutations of *EGFR* T790M and V843I were reported to confer an inherited susceptibility to lung adenocarcinoma [28, 29], which may explain at least in part the phenomenon of family lung cancers. In our previous study, Yang et al. showed that the L858R mutation is associated with polymorphisms of genes related to estrogen biosynthesis and metabolism in non-smoking female patients with lung adenocarcinoma [30]. In a genome-wide association study (GWAS) involving 584 cases and 585 controls in Taiwan, the *CLPTM1L-TERT* SNP, rs2736100, on chromosome 5p15.33 was directly associated with the risk of lung adenocarcinoma in non-smoking females [31]. Recently, we further identified the *YAP1* R331W missense mutation as an allele predisposing to lung adenocarcinoma in Taiwan with high familial penetrance [15]. As there were various *EGFR* mutation subtypes in *YAP1* carriers, other second hits may be needed to initiate lung cancer formation. Herein, it has not been possible to definitely conclude the association between *YAP1* and *EGFR* mutations because of the limited case numbers. Whether these risk genes are also involved in *EGFR* mutations requires further study.

In conclusion, our results showed that familial lung cancer patients were carrying a higher rate of *EGFR* mutations. Moreover, the distribution of *EGFR* mutation subtypes among patients with lung cancer family history was random. This phenomenon may hint at the direction of future research into lung carcinogenesis and *EGFR* mutagenesis.

## MATERIALS AND METHODS

### Patients

This was a multicenter retrospective study, which analyzed lung cancer patients with an identifiable lung cancer family history. To be eligible for the study, patients were required to have pathologically confirmed lung cancer, available tumor specimens for *EGFR* mutation testing, and identifiable family history of lung cancer. Family history of lung cancer was defined as at least one first-degree relative of the index case having this disease. Patients were excluded if they had other active malignancies, a lung tumor with doubtful origin, or uncertain family history of lung cancer.

Participants in this study came from two major patient groups and the participant selection flowchart is disclosed in Figure 1. One multicenter cohort came from a prospective study, which identified five driver mutations in lung adenocarcinoma patients in Taiwan (*n* = 1772) [7]. Another retrospective cohort included lung cancer patients with at least 2 tumor tissues available within a family and who were diagnosed and treated at Taichung Veterans General Hospital (TCVGH) and Chang Gung Memorial Hospital (CGMH) between 2002 and 2014 (*n* = 88 within 40 families), which was aimed to evaluate the “intra-family” distribution of *EGFR* mutations.

Clinical data for analysis included patients' age, gender, smoking status, tumor stage, and family relatedness. TNM (tumor, node, and metastases) staging was done according to the 7th edition of the American Joint Committee for Cancer (AJCC) staging system [32]. This study was approved by the institutional review boards of the participating institutions.

### EGFR and YAP1 mutation analysis

Tumor specimens were collected and procured for *EGFR* mutation analysis as previously described [33, 34]. The detection method used in the present study was Matrix-Assisted-Laser-Desorption-Ionization Time-of-Flight mass spectrometry (MALDI-TOF MS). The detection spectrum of MALDI-TOF MS is summarized in Supplementary Table S1. Briefly, we performed the testing according to the instructions provided by the MassARRAY^®^ system (Sequenom, San Diego, CA). With respect to the biochemical reaction, PCR was used to amplify the region containing the tyrosine kinase domain of the *EGFR* exons 18, 19, 20, and 21, then single nucleotide extension was performed with primers and corresponding detection probes to amplify the region containing each target mutation. After SpectroClean Resin clean up, samples were loaded onto the matrix of SpectroCHIP^®^ by Nanodispenser (Matrix) then analyzed by Bruker Autoflex MALDI-TOF MS. Data was collected and analyzed with Typer4 software (Sequenom, San Diego, CA). All the tests were performed by the ISO15189-certified TR6 Pharmacogenomics Lab (PGL), National Research Program for Biopharmaceuticals (NRPB), at the National Center of Excellence for Clinical Trial and Research of NTUH.

In the present study, we also evaluated the *YAP1* germline mutation in families with multiple lung cancer subjects in order to evaluate its association with *EGFR* mutations, and the detection method was MALDI-TOF MS as previously described [15].

### Statistical methods

Univariate analyses using Fisher's exact test and Student's *t-test* were performed to test the association between family history of lung cancer and patient characteristics, including *EGFR* mutation status. Herein, non-smokers were defined as individuals who had never smoked or had smoked fewer than 100 cigarettes in their lifetime. Current or former smokers status was determined on the basis of whether a subject had quit smoking for more than 1 year. Logistic regression models were performed to estimate the risk of *EGFR* mutation in lung adenocarcinoma patients with family history of lung cancer. As our cohort was not random in patient population, we developed a propensity score for lung cancer family history to control the potential confounding factors and selection bias [35]. The propensity score for lung cancer family history was determined by multivariate logistic regression analysis to evaluate both the *EGFR* mutation frequency and types. We included smoking status, gender, age, and stage for propensity score calculation. This propensity score represented the probability that a patient with lung adenocarcinoma would have family history of lung cancer. Based on the propensity score, patients with lung adenocarcinoma with family history were matched with two control patients who did not have family history of lung cancer. Using data for the propensity-matched patients, three types of conditional logistic regression models were fit. With regards to *EGFR* mutation types, we estimated the risk of exon 18 G719X, exon 19 deletions, and exon 21 L858R. The remaining mutations were grouped as “other uncommon mutations” because of the small number of cases. All statistical tests were done with SAS V.9.4 (SAS Institute, Cary, NC). All tests were two-tailed and *P value*s < 0.05 were considered significant.

## SUPPLEMENTARY MATERIAL FIGURE AND TABLE


