# Supplementary material for: Higher frequency but random distribution of EGFR mutation subtypes in familial lung cancer patients
Source: Oncotarget. 2016 Jul 19;7(33):53299–308. doi: 10.18632/oncotarget.10715 (PMC5288187; doi:10.18632/oncotarget.10715)
Supplement: Supplementary file 1 [file oncotarget-07-53299-s001.pdf]

Higher frequency but random distribution of *EGFR* mutation subtypes in familial lung cancer patients

Supplementary Materials

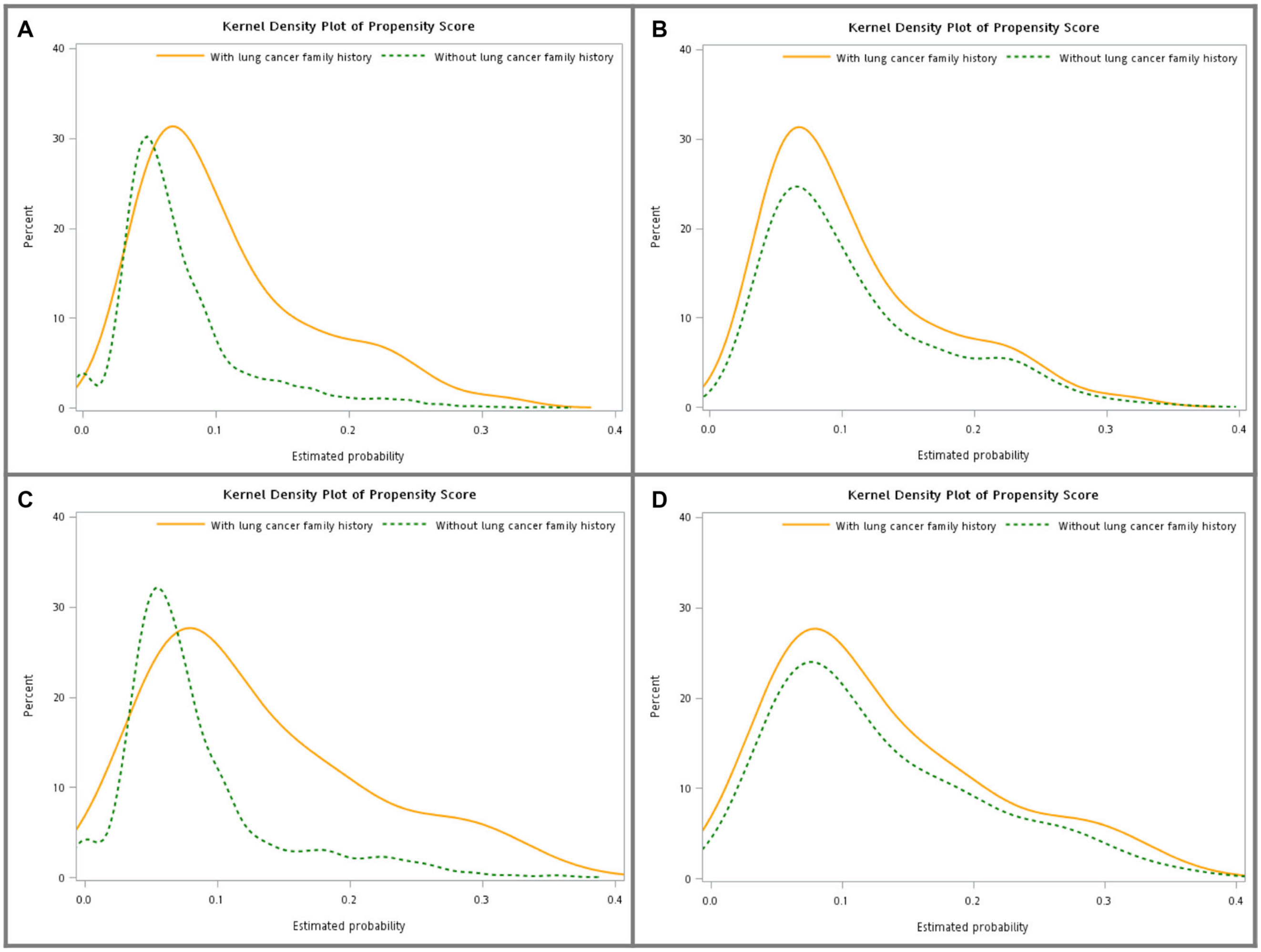

Supplementary Figure S1: Kernel density plots of propensity score before (A) and after (B) matching for familial lung adenocarcinoma patients, and before (C) and after (D) matching for *EGFR*-mutant familial lung adenocarcinoma patients.

Supplementary Table S1: *EGFR* mutations detected by MALDI-TOF MS

Exon	Mutation types (nucleic acid sequence)
18	E709A (2126A > C), E709G (2126A > G), E709V (2126A > T), G719A
	(2156G > C), G719C (2155G > T), G719N (2156G > A), G719S (2155G > A)
19	Del E746_A750 (2235_2249del15, 2236_2250del15),
	Del E746_T751 (2236_2253del18),
	Del E746_T751 > A (2237_2251del15),
	Del E746-T751 > I (2235_2252 > AAT),
	Del E746_S752 > A (2237_2254del18),
	Del E746_S752 > D (2238_2255del18),
	Del E746_S752 > V (2237_2255 > T),
	Del L747_E749 (2239_2247del9),
	Del L747_A750 > P (2238_2248 > GC, 2239_2248TTAAGAGAAG > C),
	Del L747_T751 (2239_2253del15, 2240_2254del15),
	Del L747_T751 > P (2239_2251 > C),
	Del L747_T751 > Q (2238_2252 > GCA),
	Del L747_T751 > S (2240_2251del12),
	Del L747_S752 (2239_2256del18),
	Del L747_P753 > Q (2239_2258 > CA),
	Del L747_P753 > S (2240_2257del18)
20	S768I (2303G > T), T790M (2369C > T)
21	L858Q (2573T > A), L858R (2573T > G), L861Q (2582T > A)

EGFR, epidermal growth factor receptor; MALDI-TOF MS, matrix-assisted laser desorption ionization-time of flight mass spectrometry.
